# *Ulmus pumila* Linné (Ulmi) Extract Attenuates Inflammatory Responses in Atopic Dermatitis by Modulating Lipid Peroxidation and Oxidative Stress

**DOI:** 10.3390/antiox15060683

**Published:** 2026-05-29

**Authors:** Min Jung Kim, Mi Jin Jang, Young Zoo You, Ye Jin Yang, Ji Woong Heo, Hee Ho Kim, Hun Hwan Kim, Se Hyo Jeong, Gon Sup Kim, Young Woo Kim, Ju-Hye Yang, Ryounghoon Jeon, Sang-Hyun An, Kwang Il Park

**Affiliations:** 1 College of Veterinary Medicine, Gyeongsang National University, Jinju 52828, Republic of Korea; minjung0102@gnu.ac.kr (M.J.K.); happyzoo0120@hanmail.net (Y.Z.Y.); yang93810@gnu.ac.kr (Y.J.Y.); hujiw7806@gnu.ac.kr (J.W.H.); hiho2323@gnu.ac.kr (H.H.K.); tpgy123@gnu.ac.kr (S.H.J.); gonskim@gnu.ac.kr (G.S.K.); 2 Preclinical Research Center, Daegu Gyeongbuk Medical Innovation Foundation (K-MEDI Hub), Daegu 41061, Republic of Korea; mijin22@kmedihub.re.kr (M.J.J.); rhjeon@kmedihub.re.kr (R.J.); 3 Center for Bio-Health Research, Division of Gyeongnam Bio-Environmental Research, Korea Institute of Toxicology (KIT), 17 Jeigok-gil, Jinju 52834, Republic of Korea; hunhwan.kim@kitox.re.kr; 4 College of Korean Medicine, Dongguk University, Gyeongju 38066, Republic of Korea; ywk@dongguk.ac.kr; 5 Korean Medicine (KM) Application Center, Korea Institute of Oriental Medicine, Daegu 41062, Republic of Korea; jjuhye@kiom.re.kr

**Keywords:** *Ulmus pumila* Linné, lipid peroxidation, oxidative stress, atopic dermatitis, 4-hydroxynonenal

## Abstract

Background: Atopic dermatitis (AD) is a chronic inflammatory skin disorder characterized by oxidative stress and impaired skin barrier function. These pathological features contribute to persistent inflammation and symptom exacerbation, highlighting the need for therapies that can both reduce oxidative stress and modulate inflammatory pathways. Methods: *Ulmus pumila* Linné (Ulmi) was prepared via hot water extraction and tested for cytotoxicity, antioxidant activity, and anti-inflammatory effects in HaCaT keratinocytes stimulated with tumor necrosis factor-alpha (TNF-α) and interferon-gamma (IFN-γ). In vivo efficacy was assessed using a 2,4-dinitrochlorobenzene (DNCB)-induced AD model in SKH-1 hairless mice. Bioactive compounds were identified using liquid chromatography–quadrupole time-of-flight tandem mass spectrometry (LC-QTOF-MS/MS), and molecular docking analysis was performed to evaluate the binding affinity of these compounds to aldehyde dehydrogenase 2 (ALDH2). Results: 3-(4,5-dimethylthiazol-2-yl)-2,5-diphenyltetrazolium bromide (MTT) assays confirmed that Ulmi was safe at concentrations up to 400 μg/mL. In TNF-α/IFN-γ-stimulated HaCaT cells, Ulmi significantly upregulated ALDH2 expression in a dose-dependent manner and reduced reactive oxygen species (ROS) production. The extract also suppressed pro-inflammatory mediators such as inducible nitric oxide synthase (iNOS) and cyclooxygenase-2 (COX-2), while inhibiting the activation of nuclear factor kappa B (NF-κB) and Janus kinase/signal transducer and activator of transcription (JAK/STAT) signaling pathways. In the AD mouse model, Ulmi treatment improved clinical skin scores, reduced epidermal thickness, and decreased inflammatory markers compared to untreated controls. LC-QTOF-MS/MS analysis identified eight bioactive compounds, with procyanidin B2, catechin, and epicatechin as major constituents. Molecular docking revealed that procyanidin B2 had the strongest binding affinity to ALDH2 (−9.5 kcal/mol). Conclusions: These findings demonstrate that Ulmi effectively ameliorates AD-like symptoms through ALDH2-mediated antioxidant mechanisms and anti-inflammatory effects. The results suggest that Ulmi may serve as a promising natural therapeutic agent for the management of atopic dermatitis.

## 1. Introduction

Patients diagnosed with atopic dermatitis (AD) exhibit chronic inflammatory responses, accompanied by elevated levels of oxidative stress and resultant cellular damage relative to healthy controls [1,2,3]. This is evidenced by observations including shortened telomere length and decreased blood glutathione levels. Among the diverse products generated by oxidative stress, 4-hydroxynonenal (4-HNE) is notably recognized as a highly cytotoxic aldehyde that plays a significant role in mediating cellular damage [4,5]. Furthermore, 4-HNE has been reported to aggravate inflammatory processes in AD through the enhanced production of pro-inflammatory cytokines [6].

Therapeutic strategies for AD are broadly classified into topical and systemic approaches [7]. Topical interventions include the use of emollients, corticosteroids, calcineurin inhibitors, antibiotics, and phototherapy, whereas systemic options comprise immunosuppressive agents such as corticosteroids, cyclosporine, methotrexate, mycophenolate mofetil, and azathioprine [8,9]. Despite these therapeutic options, many first-line treatments show limited efficacy and may cause significant adverse effects, particularly in pediatric populations [10,11]. Topical corticosteroids, which inhibit antigen processing and the release of inflammatory cytokines in immune cells [12,13,14], are widely used as first-line therapies for AD [15,16,17,18]. However, their prolonged use is associated with adverse effects such as skin atrophy, muscle weakness, facial edema, psoriasis, and bleeding [19,20,21], and may also result in nonspecific immunosuppression [22]. These limitations highlight the need for the development of alternative therapies for atopic conditions, which remains a global challenge. Moreover, current therapies primarily suppress inflammatory responses but do not adequately address oxidative stress-mediated mechanisms that further amplify inflammation, suggesting the importance of targeting the oxidative stress-inflammation axis [23,24].

Bioactive compounds derived from natural sources, particularly those rich in polyphenols and flavonoids [25], have attracted considerable attention as potential therapeutic agents for AD due to their anti-inflammatory activities [26,27]. In this context, several natural products, including green tea, curcumin, and propolis, have been extensively studied for their efficacy in AD, largely attributed to their antioxidant capacity and ability to modulate inflammatory responses [28]. For instance, epigallocatechin-3-gallate (EGCG) from green tea has been shown to attenuate AD-like skin inflammation by suppressing inflammatory signaling and reducing cytokine production in both in vitro and in vivo models [29]. Similarly, curcumin has demonstrated therapeutic potential by modulating JAK/STAT signaling pathways and improving skin barrier function in AD models [30], while propolis-derived compounds have been reported to alleviate AD symptoms through antioxidant and immunomodulatory effects [31]. However, these approaches mainly rely on general ROS scavenging and lack specificity in targeting underlying oxidative damage pathways. Numerous studies have demonstrated the therapeutic benefits of traditional herbal medicine for patients. *Ulmus pumila* Linné (Ulmi), which contains key characteristic components such as catechin and procyanidin B2, has been demonstrated to exert beneficial effects in the management of AD [32]. Also, in clinical studies, procyanidin B2 has been demonstrated to enhance collagen synthesis and improve skin elasticity, thereby mitigating the effects of skin aging. Several natural products have demonstrated efficacy in patients with AD without eliciting side effects [33,34,35]. Despite these promising findings, the precise mechanisms by which natural compounds modulate lipid peroxidation-derived toxic aldehydes, such as 4-HNE, remain insufficiently explored. However, the ability of Ulmi extract to mitigate oxidative stress-induced damage, specifically by detoxifying lipid peroxidation-derived aldehydes through ALDH2 activation, may represent a novel therapeutic approach for AD [36,37].

This study aims to evaluate the potential of Ulmi to attenuate ROS-induced lipid peroxidation and suppress JAK2/STAT-1- and NF-κB-mediated inflammation, with particular attention to the role of ALDH2 activation in these processes. We employed both in vitro (TNF-α/IFN-γ-stimulated HaCaT keratinocytes) and in vivo (DNCB-induced SKH-1 mouse model) experimental systems to investigate oxidative stress and inflammatory signaling pathways. Epidermal thickness was further assessed in skin tissue sections as a histological indicator of AD-like skin inflammation and tissue remodeling. Given that oxidative stress contributes to the amplification of inflammatory signaling, this study further focuses on whether modulation of ALDH2 can influence the oxidative stress-inflammation axis in AD. Therefore, we sought to verify whether Ulmi extract enhances ALDH2 activity to reduce 4-HNE accumulation. This study also aimed to assess if Ulmi effectively ameliorates 2,4-Dinitrochlorobenzene (DNCB)-induced AD-like lesions in SKH-1 mice. Our findings demonstrate that Ulmi extract significantly reduces oxidative stress and inflammatory responses while improving barrier integrity, suggesting that ALDH2 activation-mediated detoxification of lipid peroxidation products may represent a novel mechanistic strategy for AD treatment.

## 2. Materials and Methods

### 2.1. Extraction and Sampling

*Ulmus pumila* Linné was prepared as a water extract (Ulmi). The cortex of the trunk and root was carefully selected and purchased from a specialized herbal medicine market (Puremind Co., Ltd., Yeongcheon, Gyeongsangbuk-do, Republic of Korea). The plant material was authenticated, and a voucher specimen (Herbarium No. 2010-W308) was deposited. Additional information includes: manufacturing date, 2 April 2010; weight per unit, 50.00 g; final extract weight, 3.21 g; country of origin, Republic of Korea; producer/importer, Yeon-ho Kim (Yeongcheon-si, Gyeongsangbuk-do, Republic of Korea). The dried plant material was extracted with distilled water (10 × *w*/*v*) at 60 °C for 3 h, and this process was repeated three times. The combined extracts were concentrated under reduced pressure using a vacuum concentrator (EYELA, Tokyo, Japan), freeze-dried, and stored at −20 °C. The extraction yield (6.42%) was calculated as the weight of the dried extract divided by the initial dry weight of the plant material, multiplied by 100. For experiments, the lyophilized powder (100 mg) was dissolved in 1 mL of distilled water and filtered through a 0.22 μm membrane filter.

### 2.2. Animals and Experiments

Six-week-old SKH-1 mice (25–28 g) were purchased from Orient Bio (Seoul, Republic of Korea) and housed under controlled environmental conditions (22 ± 3 °C, 55 ± 5% humidity, 12 h light/dark cycle) with free access to food and water. All experimental protocols were approved by the Institutional Animal Care and Use Committee of the Daegu Gyeongbuk Medical Innovation Foundation (K-MEDI Hub) (DGMIF-19043003-00). The animals were randomly divided into three groups (n = 5 per group): (i) untreated normal control, (ii) DNCB-induced AD model control, and (iii) Ulmi-treated AD group. AD-like skin lesions were induced using DNCB, a well-established allergen that elicits immune responses resembling those observed in human AD. During week 1, 200 μL of 1% (*w*/*v*) DNCB in acetone/olive oil (3:1, *v*/*v*) was applied to the dorsal skin once daily for initial sensitization. To maintain AD-like symptoms, 0.5% (*w*/*v*) DNCB (200 μL) was applied three times per week during weeks 2–5. During weeks 6–7, Ulmi extract was topically applied to the dorsal skin at a dose of 200 mg/kg once daily, while 0.5% (*w*/*v*) DNCB (200 μL) was applied twice per week. Skin lesion severity was evaluated using a scoring system that considered multiple clinical features, including erythema, edema, xerosis, excoriation, and crusting.

### 2.3. Hematoxylin and Eosin (H&E) Staining

Mice were sacrificed at 3, 7, and 14 days after treatment, and dorsal skin samples were collected for histological analysis. The tissues were fixed in 10% formaldehyde solution (Sigma-Aldrich, St. Louis, MO, USA) for 24 h and subsequently processed for paraffin embedding. Paraffin-embedded samples were sectioned at a thickness of 4 μm and stained with hematoxylin and eosin (H&E) to examine histopathological changes, including epidermal thickness. Quantitative assessment of epidermal thickness was performed using HK Basic software (version 4.12.28926; KOPTIC, Seoul, Republic of Korea). For each mouse, three randomly chosen non-overlapping regions were analyzed, and at least five independent measurements were obtained per region, yielding a total of 15 measurements per animal. All measurements were performed in a blinded manner using a standardized protocol, and inter-rater reliability was confirmed by calculating the intraclass correlation coefficient.

### 2.4. Immunohistochemistry (IHC) Staining

For IHC, the sections were deparaffinized, rehydrated, and incubated in water. The sections were then treated with 0.3% (*v*/*v*) hydrogen peroxide (H_2_O_2_) to inhibit peroxidase activity for 5 min and blocked with protein blocking serum for 1 h. And then, the sections were incubated overnight at 4 °C with the primary antibody against 4HNE (ab46545; Abcam, Waltham, MA, USA) at a dilution of 1:100. After rinsing with PBS, the sections were incubated with a biotin-conjugated secondary antibody (1:200) for one hour at room temperature. For detection, sections were incubated with the ABC complex kit (Vector Laboratories, Burlingame, CA, USA) for 30 min and subsequently visualized using the Vector NovaRED substrate kit (Vector Laboratories, Burlingame, CA, USA). The nuclei were counterstained using hematoxylin.

### 2.5. Cell Culture Maintenance

HaCaT cells (human keratinocytes) were obtained from the Korea Institute of Oriental Medicine and originally sourced from Cell Lines Service (Eppelheim, Germany). Cells were maintained in Dulbecco’s Modified Eagle’s Medium supplemented with 10% heat-inactivated fetal bovine serum and 1% penicillin/streptomycin (Thermo Fisher Scientific, Waltham, MA, USA). Prior to experimental use, the cells were subcultured for at least three passages to ensure stable growth characteristics and to minimize variability associated with early passage conditions. Based on cell viability assessment, concentrations that did not induce cytotoxicity were selected for all subsequent experiments. Ulmi was applied at 100 and 200 μg/mL, while dexamethasone (Dexa) was used as a positive control at 10 μg/mL to confirm anti-inflammatory effects.

### 2.6. HaCaT Cell Viability

Cell viability was determined using the MTT assay (#298-93-1, Duchefa Biochemie, Haarlem, The Netherlands). The cells (1 × 10^4^ per well) were seeded into 96-well plates and allowed to stabilize for 24 h. Ulmi concentrations were selected based on a wide preliminary range-finding design to evaluate potential cytotoxicity across low-to-high exposure levels. HaCaT cells were exposed to Ulmi at concentrations ranging from 0 to 400 µg/mL for 24 h. After treatment, MTT solution (0.5 mg/mL in PBS) was added to each well and incubated for 2 h at 37 °C. The culture medium was then removed, and the formed formazan crystals were dissolved in dimethyl sulfoxide (DMSO). The absorbance was then measured with a microplate reader (Agilent Technologies, Santa Clara, CA, USA) set at 565 nm.

### 2.7. Evaluation of Intracellular ROS Production by DCF-DA Assay in HaCaT Cell

HaCaT cells were seeded in 6-well plates at 5 × 10^5^ cells per well and stabilized for 24 h at 37 °C under 5% CO_2_. Intracellular ROS levels were measured using 2′,7′-dichlorofluorescein diacetate (DCF-DA; Thermo Fisher Scientific, Waltham, MA, USA), which was prepared at 10 μM and incubated with cells for 1 h. Cells were then treated with Ulmi at concentrations of 100 and 200 μg/mL for 24 h, and dexa (10 μg/mL) was used as a positive control under the same conditions. Fluorescence intensity was analyzed using a microplate reader and a Cytation 7 imaging system (Agilent Technologies, Santa Clara, CA, USA) with excitation at 485 nm and emission at 535 nm [38].

### 2.8. Protein Extraction and Western Blotting

Protein levels in extract-treated cells were subsequently assessed. HaCaT cells were initially seeded at 1 × 10^6^ cells per 60 mm dish and allowed to stabilize for 24 h. After stabilization, cells were co-treated with Ulmi (100 and 200 μg/mL), TNF-α (10 ng/mL; NKMAX, Seongnam, Republic of Korea), and IFN-γ (10 ng/mL; NKMAX, Seongnam, Republic of Korea) and incubated for 24 h. Cells were washed three times with PBS (BioSeSang, Seoul, Republic of Korea) and lysed in RIPA buffer (Thermo Fisher Scientific, Waltham, MA, USA) supplemented with a protease inhibitor cocktail (100×, GenDEPOT, Katy, TX, USA). The lysates were centrifuged at 13,000 rpm for 15 min at 4 °C, and the clarified supernatants were collected. Protein concentrations were determined using a bicinchoninic acid (BCA) assay kit (Bio-Max, Seoul, Republic of Korea). For electrophoresis, 20 μg of protein from each sample was mixed with 5 × sample buffer (ELPis, Daejeon, Republic of Korea), denatured at 80 °C for 15 min, and separated on SDS-PAGE gels. The resolved proteins were transferred onto PVDF membranes (GVS Filter Technology, Sanford, ME, USA), followed by blocking with 5% bovine serum albumin in TBS containing 0.1% (*v*/*v*) Tween-20 (TBS-T) for 2 h at room temperature. The membranes were incubated overnight at 4 °C with primary antibodies against ALDH2 (#ATGA0450, NKMAX, Seoul, Republic of Korea), p-NF-κB (#3031, Cell Signaling Technology, Danvers, MA, USA), p-IκBα (#2859, Cell Signaling Technology, Danvers, MA, USA), COX-2 (#12282, Cell Signaling Technology, Danvers, MA, USA), iNOS (#13120, Cell Signaling Technology, Danvers, MA, USA), p-JAK2 (SC-21870, Santa Cruz Biotechnology, Santa Cruz, CA, USA), p-STAT1 (#ATGA0250, NKMAX, Seoul, Republic of Korea), and GAPDH (#2118, Cell Signaling Technology, Danvers, MA, USA), all used at a dilution of 1:1000. After washing with TBS-T, membranes were incubated with HRP-conjugated secondary antibodies (goat anti-mouse IgG, #1706516; goat anti-rabbit IgG, #1706515; Bio-Rad, Hercules, CA, USA) for 2 h at room temperature. Protein bands were visualized using a SuperSignal^TM^ West Pico PLUS chemiluminescent substrate (Thermo Fisher Scientific, Seoul, Republic of Korea) and detected with an imaging system (Shenhua Science Technology, Hangzhou, China). Densitometric analysis was performed using ImageJ software (version 1.54g; NIH, Bethesda, MD, USA), and protein expression levels were normalized to GAPDH, which remained stable across all experimental conditions.

### 2.9. Antioxidant Capacity Analysis

For the DPPH assay, the working solution was prepared in methanol and adjusted to an absorbance of 1.00 ± 0.10 at 517 nm. Samples were diluted to various concentrations with methanol, and 100 μL of each sample was mixed with 100 μL of the DPPH solution. The reaction mixtures were incubated at 37 °C in the dark for 30 min, after which absorbance was measured at 517 nm using a microplate spectrophotometer. Methanol was used as a control in place of the sample. For the ABTS assay, ABTS (7.4 mM) and potassium persulfate (2.6 mM) were mixed at a 1:1 ratio and incubated overnight in the dark at room temperature to generate ABTS^+^ radicals. The resulting solution was diluted with distilled water to an absorbance of 0.70 ± 0.02 at 734 nm. Then, 100 μL of each sample was combined with 100 μL of the ABTS^+^ solution, and absorbance was recorded at 734 nm. Distilled water was used as the control. Ascorbic acid (100 μg/mL) was used as a positive control in both assays. The concentrations used in the DPPH and ABTS assays (10–300 μg/mL) were selected to evaluate dose-dependent antioxidant activity within the measurable dynamic range of the assays.

### 2.10. Total Polyphenol and Flavonoid Content

The total phenolic and flavonoid levels in Ulmi were assessed by employing the Folin–Ciocalteu assay and the aluminum chloride colorimetric method, respectively. For polyphenol content measurement, gallic acid (Sigma-Aldrich, St. Louis, MO, USA, Lot # 1003409474, purity ≥97.5%) solutions (1 mg/mL in distilled water) were prepared and serially diluted to construct a calibration curve. A mixture was prepared comprising 100 μL of Ulmi (2 mg/mL) or gallic acid standard, 500 μL of 2N Folin’s phenol reagent (Sigma-Aldrich, St. Louis, MO, USA), and 400 μL of 7.5% sodium carbonate (Na_2_CO_3_). After mixing thoroughly, the mixtures were kept in the dark at room temperature for 1 h. Absorbance was recorded at 750 nm with a SYNERGY H1 microplate reader (BioTek, Winooski, VT, USA). The results were expressed as mg gallic acid equivalents (GAE) per g, according to the calibration curve. For flavonoid content measurement, quercetin (Sigma-Aldrich, St. Louis, MO, USA, Lot # 102549872, purity ≥ 95%) solutions (1 mg/mL in methanol) were prepared and diluted to establish a calibration curve. A mixture of 100 μL Ulmi (2 mg/mL) or quercetin standard, 860 μL of 80% ethanol, 20 μL of 10% aluminum chloride (AlCl_3_), and 20 μL of 1 M potassium acetate (BIOSTEM, Seoul, Republic of Korea) was prepared. The mixtures were incubated at room temperature for 40 min, and absorbance was read at 415 nm with the SYNERGY H1 microplate reader. The outcomes were presented as mg quercetin equivalents (QE) per g. All standard compounds were of analytical grade, stored under manufacturer-recommended conditions, and freshly prepared prior to use to ensure reliability and reproducibility of the calibration curves.

### 2.11. LC-QTOF-MS/MS Analysis Conditions

Phytochemical profiling of Ulmi was performed using a Nexera XS UHPLC system (Shimadzu, Kyoto, Japan) coupled with an X500R QTOF-MS (SCIEX ExionLCAD system, Framingham, MA, USA) equipped with an electrospray ionization (ESI) source. Chromatographic separation was conducted on a Pronto SIL 120-5-C18 SH column (150 × 4.6 mm, 5 μm; Bischoff Chromatography, Leonberg, Germany) maintained at 35 °C. The mobile phases consisted of 0.1% formic acid in water (A) and 0.1% formic acid in acetonitrile (B). Elution was carried out using a gradient program starting at 5% B, which was held constant for the initial 10 min. The proportion of B was then increased to 20% over the next 10 min, followed by a gradual rise to 25% at 30 min. Subsequently, B was ramped to 40% by 40 min and further elevated to 70% at 55 min. The gradient reached 95% B at 70 min, before returning to 10% B at 75 min for re-equilibration. The flow rate was set at 0.5 mL/min, and the injection volume was 2 μL. Mass spectrometric detection was performed in positive ion mode using ESI with information-dependent acquisition (IDA). The ion spray voltage was set to 5500 V, while the ion source gases (GS1 and GS2) were both maintained at 50 psi, and the source temperature was 550 °C. The TOF-MS scan range was m/z 100–1500, and MS/MS data were acquired over m/z 50–1500. The declustering potential (DP) was 80 V, and the collision energy (CE) was applied at 35 ± 15 V. Data acquisition and compound identification were carried out using SCIEX OS software (version 3.0.0.3339).

### 2.12. Molecular Docking Analysis

Molecular docking was conducted with the ALDH2 protein structure (PDB ID: 8DR9) retrieved from the Protein Data Bank. The three-dimensional structures of the compounds were downloaded from PubChem. The compounds used in this study were catechin (CID: 9064), epicatechin (CID: 72276), procyanidin B2 (CID: 122738), proline (CID: 145742), cephaeline (CID: 442195), mangiferin (CID: 5281647), 6-hydroxyluteolin 7-O-glucoside (CID: 185766), and atractylenolide III (CID: 155948). Docking simulations were carried out with UCSF Chimera software (version 1.18; Resource for Biocomputing, Visualization, and Informatics, University of California, San Francisco, CA, USA) (exhaustiveness = 8, binding modes = 9, maximum energy difference = 3 kcal/mol). Docking outcomes were visualized with Discovery Studio software (version 25.1.0.24284; BIOVIA, San Diego, CA, USA). Binding affinities were assessed based on the predicted free binding energy and total interaction energy. Each docking experiment was repeated three times for consistency.

### 2.13. Statistical Analysis

All experiments were performed using at least three independent biological replicates (n = 3), and results are presented as mean ± SEM. Statistical evaluations were conducted using GraphPad Prism software (version 8.0; GraphPad Software, San Diego, CA, USA). Prior to analysis, data distribution and variance homogeneity were verified, after which differences among groups were assessed by one-way analysis of variance (ANOVA). When significant effects were observed, Tukey’s multiple comparison test was applied as a post hoc procedure with appropriate adjustment for multiple testing. For in vitro studies, comparisons were made relative to the TNF-α/IFN-γ-treated group, whereas in vivo data were analyzed against the DNCB-induced group. Statistical significance was defined at *p* < 0.05.

## 3. Results

### 3.1. Effect of Ulmi on Skin Inflammation, Clinical Skin Index, Epidermal Thickness, and Inflammatory Response in DNCB-Induced SKH-1 Mice

**Figure 1:**
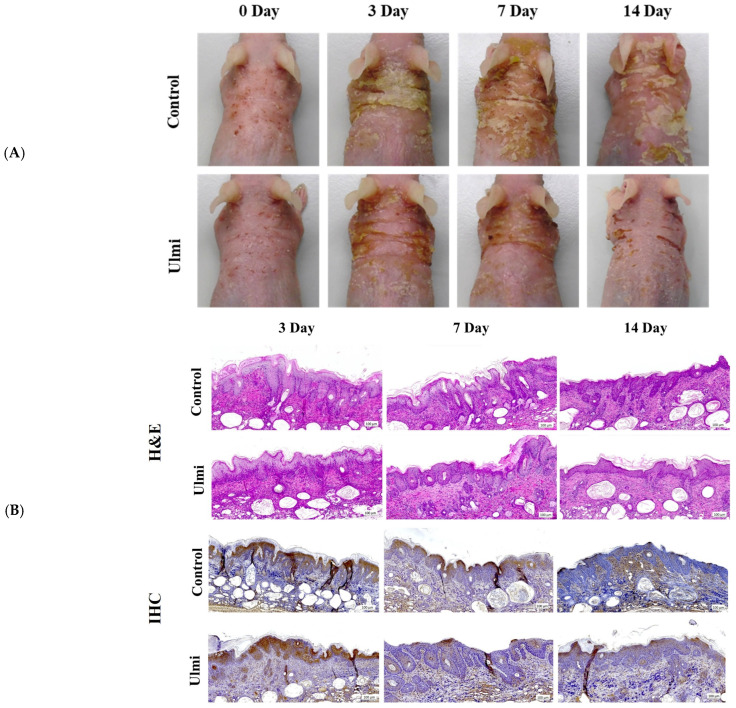

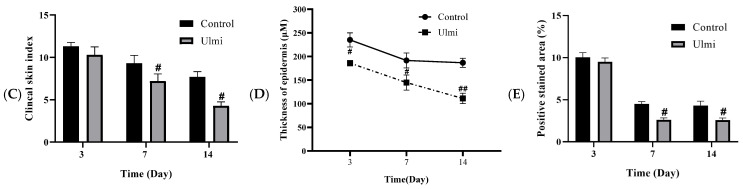
Effects of Ulmi on DNCB-induced AD, epidermal thickness, and 4-HNE accumulation in dorsal skin tissue of SKH-1 mice. (**A**) Representative clinical skin features of each experimental group. (**B**) Representative H&E staining and immunohistochemical staining of 4-HNE in dorsal skin tissue. (**C**) Clinical skin severity index (n = 5). (**D**) Epidermal thickness. (**E**) Quantification of 4-HNE positive area (%). Ulmi extract was administered at a dose of 200 mg/kg (topical application, once daily). # *p* < 0.05, ## *p* < 0.01 vs. normal control group. Scale bar: 100 µm.

To evaluate the therapeutic effects of Ulmi on dermatitis-like skin inflammation, Ulmi extract (200 mg/kg) was topically applied once daily during weeks 6–7. We assessed both the visual appearance of skin lesions and the clinical skin index over a 14-day period. As shown in Figure 1A, mice in the AD model control group developed progressive erythema, scaling, and thickened skin that persisted throughout the experiment. In contrast, the Ulmi-treated group exhibited visibly alleviated skin symptoms, with reduced erythema and scaling observed as early as day 7, and substantial improvement by day 14. Quantitative assessment of skin severity confirmed these observations (Figure 1C). On day 3, there was no significant difference between the groups. However, by day 7, the clinical skin index was significantly decreased in the AD model control group compared to the normal control group (*p* < 0.05). This reduction was further pronounced on day 14, where Ulmi administration led to a marked improvement in clinical scores (*p* < 0.05).

The anti-inflammatory and barrier-protective effects of Ulmi were assessed in AD-induced SKH-1 mouse skin through histological analysis, including H&E staining and immunohistochemistry (IHC). Compared with the AD model control group, mice treated with Ulmi showed a significant decrease in epidermal thickness, indicating an overall improvement in skin integrity. Measurements taken on days 3, 7, and 14 revealed a time-dependent decline in epidermal thickness following Ulmi treatment. These results indicate that Ulmi mitigates epidermal hyperplasia and attenuates inflammatory changes. In addition, IHC analysis revealed decreased expression of oxidative stress-related markers (4-HNE) in the Ulmi-treated group, suggesting that Ulmi effectively suppresses oxidative injury (Figure 1B,E). Quantitative analysis further demonstrated that the Ulmi-treated group showed significantly lower epidermal thickness than controls (*p* < 0.05), with notable differences observed from day 3 (Figure 1D). Especially on day 14, the epidermal thickness was significantly reduced (*p* < 0.01). Collectively, these findings demonstrate that Ulmi alleviates inflammatory responses and contributes to restoration of the skin barrier in the AD model control group.

### 3.2. Ulmi Activates ALDH2 in TNF-α/IFN-γ Induced Oxidative Stress Conditions

**Figure 2:**
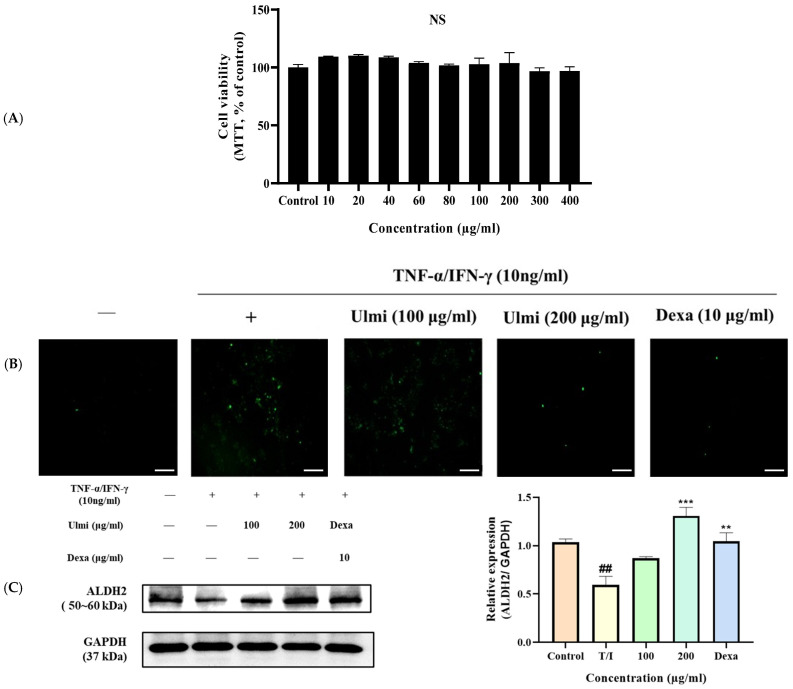
Effect of Ulmi on cell viability, ALDH2 expression, and ROS production in TNF-α/IFN-γ-stimulated HaCaT cells. (**A**) Cell viability was assessed by MTT assay after treatment with Ulmi at the indicated concentrations for 24 h. (**B**) DCF-DA fluorescence microscopy images showing ROS levels in HaCaT cells treated with TNF-α/IFN-γ (10 ng/mL each), Ulmi, and Dexa. (**C**) ALDH2 protein expression measured by Western blotting. The bar graph represents densitometric quantification of ALDH2 normalized to GAPDH (mean ± SEM, n = 3). Statistical significance indicated by *** *p* < 0.001, ** *p* < 0.01 vs. T/I group; ## *p* < 0.01 vs. control group. NS, not significant. Scale bar: 200 µm.

The cytotoxic effects of Ulmi on HaCaT cells were evaluated using an MTT assay. Across the tested concentration range (10–400 μg/mL), Ulmi did not exhibit any detectable cytotoxicity. These findings indicate that Ulmi is non-toxic under the experimental conditions employed, supporting its use at concentrations up to 400 μg/mL in subsequent experiments (Figure 2A). In addition, ROS production was increased by TNF-α/IFN-γ stimulation, whereas treatment with Ulmi (100 and 200 μg/mL) or Dexa markedly reduced ROS levels (Figure 2B). To evaluate the antioxidant potential of the Ulmi extract, we measured the expression of ALDH2 in HaCaT cells stimulated with TNF-α/IFN-γ. As shown in Figure 2C, stimulation with TNF-α/IFN-γ significantly decreased ALDH2 expression (*p* < 0.01) compared to the untreated control group. However, treatment with the Ulmi extract effectively restored ALDH2 levels in a concentration-dependent manner, with the highest dose producing a statistically significant upregulation of ALDH2 (*p* < 0.001). In HaCaT cells stimulated with TNF-α/IFN-γ, Dexa (dexamethasone, used as a positive control) treatment significantly restored ALDH2 expression (*p* < 0.01). These results indicate that the Ulmi extract alleviates oxidative stress through ROS production and modulation of ALDH2.

### 3.3. Ulmi Extract Attenuates Pro-Inflammatory Mediator Expression via Inhibition of NF-κB and JAK/STAT Pathway Activation in TNF-α/IFN-γ-Stimulated HaCaT Cells

**Figure 3:**
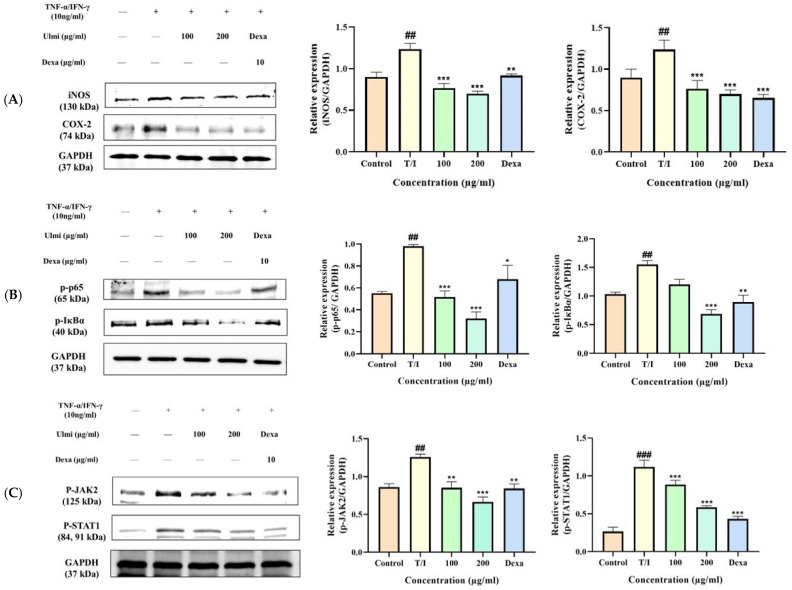
Ulmi extract suppresses pro-inflammatory mediator expression by inhibiting NF-κB and JAK/STAT pathway activation in TNF-α/IFN-γ-stimulated HaCaT cells. HaCaT cells were stimulated with TNF-α and IFN-γ (10 ng/mL each) for 24 h, followed by treatment with different concentrations of Ulmi. (**A**) The expression levels of iNOS and COX-2 were analyzed by Western blotting. (**B**) Activation of the NF-κB signaling pathway was evaluated by examining the phosphorylation status of IκBα and p65 in cells co-treated with TNF-α/IFN-γ and Ulmi. (**C**) JAK/STAT pathway activation was assessed by analyzing the phosphorylation levels of JAK2 and STAT1 by Western blotting in HaCaT cells treated with TNF-α/IFN-γ and Ulmi. Densitometric analysis was performed, and the protein expression levels were normalized to GAPDH. Results are expressed as mean ± SEM (n = 3). Statistical significance indicated by * *p* < 0.05, ** *p* < 0.01, *** *p* < 0.001 vs. T/I group; ## *p* < 0.01, ### *p* < 0.001 vs. control group.

To determine the anti-inflammatory effects of the Ulmi extract, we evaluated the expression of iNOS and COX-2 in TNF-α/IFN-γ-stimulated HaCaT cells. As shown in Figure 3A, stimulation with TNF-α/IFN-γ significantly upregulated both iNOS and COX-2 protein levels compared to the unstimulated control, indicating an induced inflammatory response (*p* < 0.01). Treatment with the Ulmi extract reduced the expression of both proteins in a concentration-dependent manner. The highest dose led to a marked suppression of iNOS and COX-2 levels (*p* < 0.001), demonstrating the extract’s potential to attenuate inflammation through downregulation of pro-inflammatory mediators.

Ulmi treatment significantly suppressed the activation of the NF-κB pathway in HaCaT cells stimulated with TNF-α/IFN-γ, as demonstrated by reduced phosphorylation levels of p65 and IκBα relative to the TNF-α/IFN-γ-only group. The reduction was dose-dependent, with 200 (*p* < 0.001) μg/mL exhibiting greater inhibition than 100 μg/mL, and the effect was comparable to that of the positive control, Dexa (Figure 3B). Similarly, Ulmi inhibited the activation of the JAK/STAT pathway, as evidenced by decreased phosphorylation of JAK2 and STAT1 in TNF-α/IFN-γ-treated HaCaT cells. Phosphorylation levels decreased significantly at both 100 and 200 (*p* < 0.001) μg/mL concentrations of Ulmi, demonstrating a dose-dependent effect and comparable inhibition to Dexa (Figure 3C). These results suggest that Ulmi effectively inhibits the pro-inflammatory signaling pathways NF-κB and JAK/STAT in keratinocytes under TNF-α/IFN-γ-induced inflammatory conditions, thereby highlighting its potential as an anti-inflammatory agent through the downregulation of pro-inflammatory protein expression.

### 3.4. TPC, TFC, Antioxidant of Ulmi

**Figure 4:**
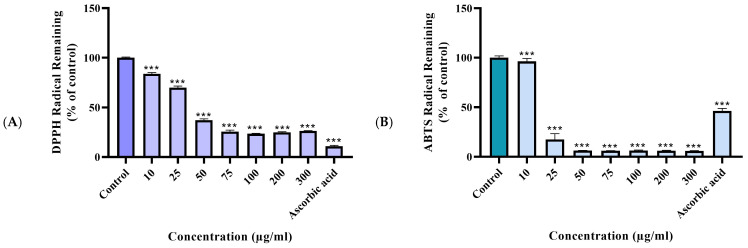
In vitro antioxidant activity of Ulmi extract evaluated by DPPH and ABTS assay. (**A**) DPPH radical scavenging activity of Ulmi extract at various concentrations (10–300 µg/mL) compared to control. Ulmi significantly reduced DPPH radical levels in a dose-dependent manner. (**B**) ABTS cation radical scavenging activity of Ulmi extract at different concentrations (10–300 µg/mL) compared to control. Ulmi showed strong dose-dependent inhibition of ABTS radicals. Data represents the mean ± SEM. Statistical significance indicated by *** *p* < 0.001 vs. control group. Ascorbic acid: AA.

To assess the antioxidant potential of Ulmi, the total contents of polyphenols and flavonoids were quantified. The total polyphenol content in Ulmi was determined to be 105.955 ± 0.003 mg GAE/g, whereas the total flavonoid content was 57.099 ± 0.400 mg QE/g (Table 1). The antioxidant effects of Ulmi were evaluated using DPPH and ABTS assays, with 100 μg/mL ascorbic acid (AA) serving as the positive control. Our results demonstrated that Ulmi, starting at 10 μg/mL, exhibited a strong antioxidant effect (*p* < 0.001) (Figure 4A,B).

### 3.5. LC-QTOF-MS-MS Analysis of Ulmi Components

**Figure 5:**
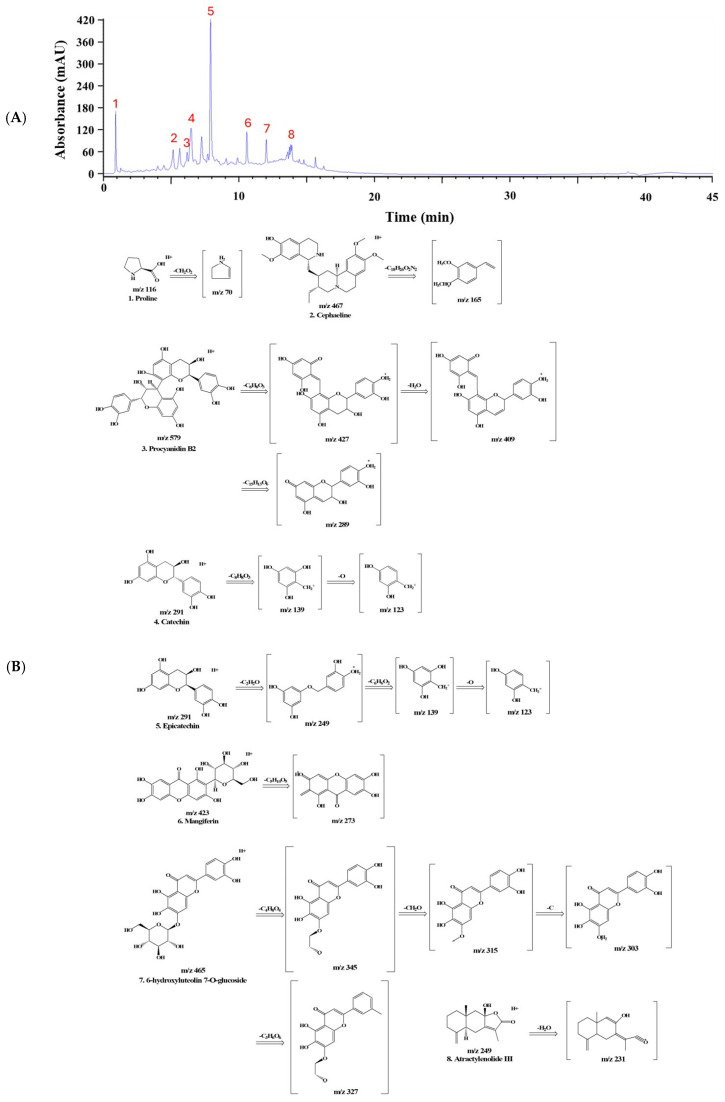
Chromatography Quadrupole Time-of-Flight Mass Spectrometry (LC-QTOF-MS-MS) analysis of Ulmi. (**A**) The chromatogram illustrates the retention times of key compounds at 284 nm. Red numbers indicate peak numbers. (**B**) while the mass spectra depict their corresponding fragmentation patterns.

The UPLC-QTOF-MS-MS analysis identified key bioactive compounds present in Ulmi. The eight compounds were tentatively identified based on the precursor ion m/z values and corresponding MS/MS fragmentation patterns, with reference to previous literature and database matching. Eight distinct compounds Proline (Amino acid), Cephaeline, Procyanidin B2, Catechin, Epicatechin, Mangiferin, 6-hydroxyluteolin 7-O-glucosid, Atractylenolide III were successfully identified (Peaks 1–8, Figure 5A). These compounds were identified through UPLC-QTOF-MS/MS profiling and should be distinguished from peaks observed in UPLC chromatograms, which reflect overall extract composition rather than compound identification. Among these, Procyanidin B2 (C_30_H_26_O_12_), Catechin (C_15_H_14_O_6_), and Epicatechin (C_15_H_14_O), recognized as indicator compounds of Ulmi, were identified (Table 2). Their molecular structures and molecular structure fragmentation are depicted in Figure 5B.

### 3.6. Molecular Docking Analysis of UImi-Derived Compounds with ALDH2

**Figure 6:**
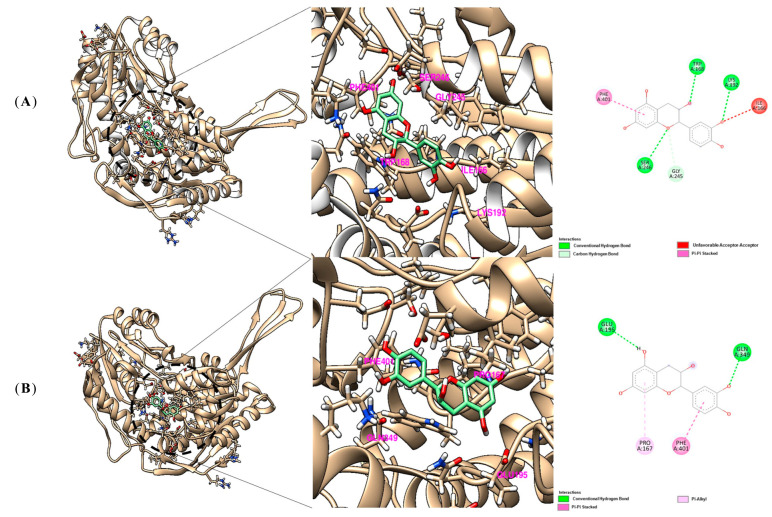

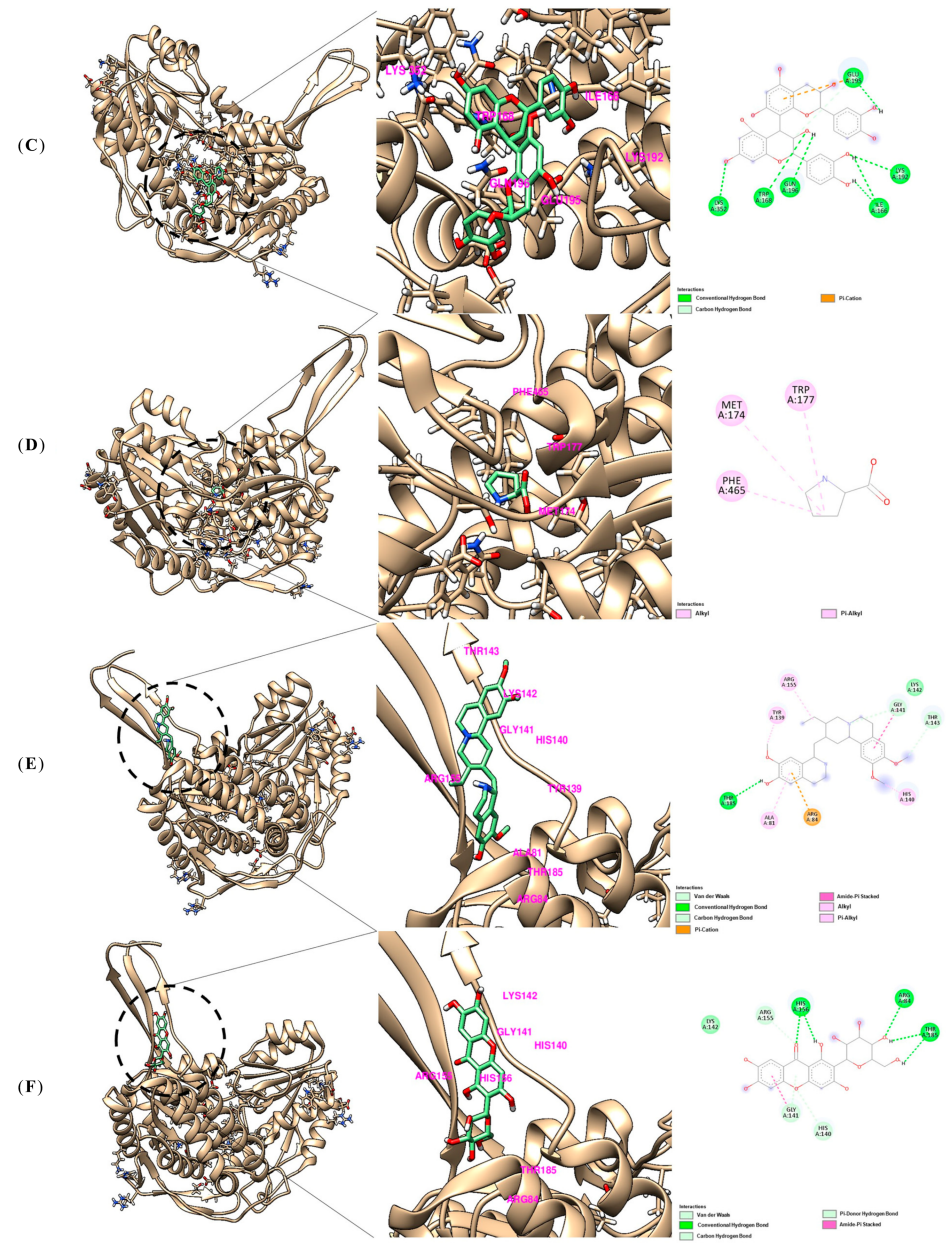

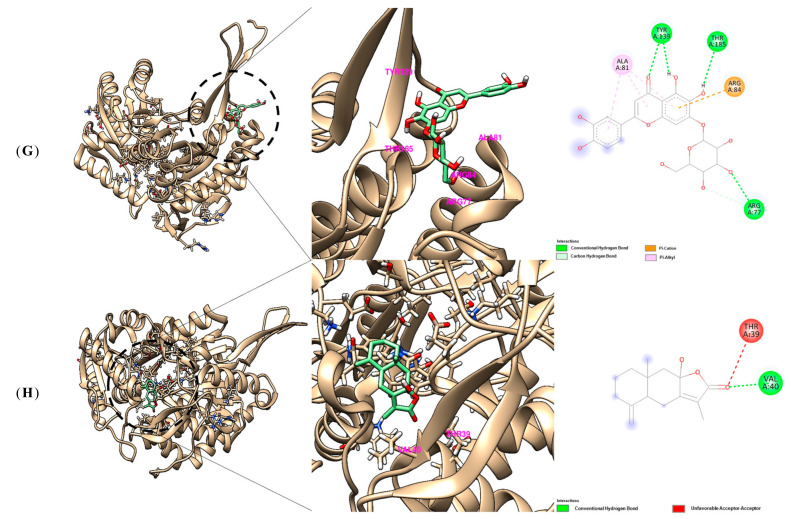
Molecular docking analysis of Ulmi-derived compounds with ALDH2. Docking structure of (**A**) Proline, (**B**) Cephaeline, (**C**) Procyanidin B2, (**D**) Catechin (**E**) Epicatechin, (**F**) Mangiferin, (**G**) 6-hydroxyluteolin 7-O-glucoside, and (**H**) Atractylenolide III with the active site of ALDH2.

A molecular docking assay was conducted to evaluate the binding affinities of Ulmi-derived compounds for ALDH2. All compounds were accommodated within the enzyme’s active site and interacted with key amino acid residues (Table 3). Among them (Figure 6), procyanidin B2 demonstrated the highest binding affinity (−9.5 kcal/mol). Similarly, 6-hydroxyluteolin 7-O-glucoside (−8.9 kcal/mol), epicatechin (−8.2 kcal/mol), and mangiferin (−8.2 kcal/mol) also demonstrated strong bindings. Notably, catechin and cephaeline both bound with comparable affinities (−7.9 kcal/mol), whereas proline showed the least favorable interaction (−4.8 kcal/mol). These findings suggest that polyphenolic compounds, especially procyanidin B2 and 6-hydroxyluteolin 7-O-glucoside, may serve as potent ALDH2 modulators through stable and high-affinity interactions with the enzyme’s active site.

## 4. Discussion

The antioxidant and anti-inflammatory properties of Ulmi were investigated using keratinocyte models exposed to TNF-α/IFN-γ-induced oxidative stress, as well as in an AD mouse model employing SKH-1 mice. Our findings indicate that Ulmi mediates its therapeutic effects through the modulation of oxidative stress-related pathways, the suppression of inflammatory mediator expression, and the restoration of skin barrier integrity.

The in vivo results from the AD-induced SKH-1 mouse model further supported these findings. Mice treated with Ulmi exhibited significant improvements in clinical skin lesions, including reductions in erythema, scaling, and thickening, compared to the control group (Figure 1A). The clinical skin index showed a significant decrease from day 7 onwards (Figure 1C), and histological analysis confirmed reduced epidermal thickness (Figure 1B,D). Furthermore, IHC results demonstrated decreased expression of 4-HNE in Ulmi-treated skin tissue, suggesting attenuation of lipid peroxidation-associated damage (Figure 1B,E). These results also imply that Ulmi may exert barrier-protective effects by regulating key components such as filaggrin and ceramide metabolism, thereby improving epidermal integrity, as supported by previous studies. *Ulmus pumila* extracts have been reported to exhibit antioxidant and anti-inflammatory properties, which are relevant to skin protection [39,40]. While direct evidence for skin regeneration remains limited, these properties suggest their potential application in dermatological and cosmetic fields. Unlike previously reported natural products or antioxidant-rich formulations that primarily improve barrier function through anti-inflammatory effects or direct moisturization [28], Ulmi may exert a distinct mechanism by enhancing aldehyde detoxification via ALDH2-related pathways. Polyphenolic constituents such as procyanidin B2 and catechin identified in this study are predicted to interact with ALDH2 and may contribute to maintaining lipid integrity under oxidative stress conditions.

This mechanism is particularly relevant in AD, where oxidative stress is increasingly recognized not only as a consequence of inflammation but also as a driver of barrier dysfunction and immune dysregulation [41]. Lipid peroxidation products such as 4-HNE function as both biomarkers and mediators of disease severity [42]. In this study, 4-HNE levels were quantitatively assessed based on IHC analysis, showing a significant reduction following Ulmi treatment, which supports the attenuation of lipid peroxidation-associated damage. This emphasizes the importance of targeting aldehyde-mediated damage rather than focusing solely on ROS [43]. In vitro, Ulmi extract markedly increased the expression of ALDH2, a crucial enzyme responsible for reactive aldehyde detoxification and the maintenance of mitochondrial redox homeostasis. The restoration of ALDH2 levels, accompanied by a reduction in ROS production (Figure 2C), indicates that Ulmi mitigates oxidative stress by enhancing endogenous detoxification mechanisms. These findings align with previous studies demonstrating that polyphenols modulate Nrf2/ARE signaling and aldehyde detoxification pathways [44,45,46,47,48].

In addition, Ulmi extract significantly inhibited pro-inflammatory mediators, including iNOS and COX-2, in HaCaT cells (Figure 3A), and suppressed NF-κB and JAK2/STAT1 signaling pathways (Figure 3B,C), consistent with previous reports [49,50]. Notably, the inhibitory effects of Ulmi at higher concentrations were comparable to those of Dexa, a widely used corticosteroid [51], suggesting its significant therapeutic potential as a natural anti-inflammatory agent with fewer expected side effects.

Phytochemical analysis further clarified the underlying mechanisms of these biological effects [52]. Ulmi was found to be abundant in polyphenols and flavonoids, compounds renowned for their antioxidant and anti-inflammatory properties [53,54,55]. LC-QTOF-MS/MS analysis tentatively identified eight major compounds, including procyanidin B2 [56], catechin [57], epicatechin [58,59], mangiferin, and 6-hydroxyluteolin 7-O-glucoside, several of which are recognized as bioactive constituents in herbal medicine (Figure 5). Among these, procyanidin B2, catechin, epicatechin, mangiferin, and 6-hydroxyluteolin 7-O-glucoside can be classified as flavonoids or flavonoid-derived polyphenols, suggesting that flavonoid-type compounds represent a major proportion of the bioactive constituents in Ulmi extract [60].

The determination of total phenolic and flavonoid contents was performed to estimate the overall abundance of antioxidant-related phytochemicals in the extract, as these compounds are widely recognized to contribute to both oxidative stress regulation and inflammatory responses [61]. In the context of AD, phenolic and flavonoid compounds have been reported to modulate key signaling pathways involved in inflammation, including NF-κB and JAK/STAT, as well as to attenuate oxidative damage [62,63,64]. Although individual contributions were not quantified, structural features of flavonoids, such as hydroxylation and polymerization, are known to influence antioxidant and anti-inflammatory activities [65]. Accordingly, catechin, epicatechin, and procyanidin B2 are likely to contribute to the observed effects, which may result from their combined action rather than a single dominant compound.

Molecular docking analysis showed strong binding affinities of these compounds to ALDH2, with procyanidin B2 and 6-hydroxyluteolin 7-O-glucoside exhibiting stable interactions (Figure 6). However, these results provide supportive in silico evidence for ALDH2-related interactions but do not confirm direct enzymatic activation. Compared with conventional antioxidant approaches that focus on ROS scavenging, Ulmi may provide a broader effect by simultaneously modulating aldehyde detoxification and inflammatory signaling pathways. This multi-target profile is advantageous given the close interplay between oxidative stress and inflammation in AD.

Collectively, our study demonstrates that Ulmi extract exerts multi-target protective effects against skin inflammation and oxidative stress by (i) modulating ALDH2-related pathways, (ii) suppressing NF-κB [66] and JAK/STAT-mediated [67,68,69] inflammatory signaling, and (iii) enhancing epidermal barrier integrity in an AD-like mouse model. This suggests a mechanism distinct from conventional antioxidants, involving the regulation of mitochondrial redox homeostasis and aldehyde-induced cytotoxicity. While our findings suggest that ALDH2 may be involved in linking oxidative stress and inflammatory responses, this should be considered a potential mechanistic pathway, and further quantitative and functional studies are required to validate this axis in AD.

Compared with current therapies such as corticosteroids, biologics, and JAK inhibitors, Ulmi may offer a complementary multi-target approach with potential advantages in cost-effectiveness and mechanistic coverage, although further studies are required to validate its clinical efficacy. Thus, Ulmi and its bioactive polyphenolic constituents represent promising candidates for the development of novel therapeutic strategies targeting inflammatory skin diseases, including AD.

## 5. Conclusions

Our findings suggest that Ulmi extract alleviates inflammatory skin conditions by simultaneously enhancing antioxidant defenses and suppressing oxidative stress-induced lipid peroxidation, as evidenced by reduced 4-HNE accumulation and increased ALDH2 expression. These effects were accompanied by inhibition of NF-κB and JAK2/STAT1 signaling pathways, leading to decreased expression of pro-inflammatory mediators in keratinocyte models. Furthermore, Ulmi treatment significantly improved clinical skin lesions and reduced epidermal thickness in an AD-like SKH-1 mouse model, indicating attenuation of inflammatory skin pathology. This integrated mechanism highlights the potential of Ulmi-derived polyphenolic compounds as multi-target natural therapeutic agents for the prevention and treatment of atopic dermatitis and related inflammatory skin disorders.

## Figures and Tables

**Table 1:** Total phenolic and flavonoid contents of crude extract.

	Items	Concentration
Ulmi	Total polyphenol (GAE mg/g) ^1^	105.955 ± 0.003
	Total flavonoid (QE mg/g) ^2^	57.099 ± 0.400

^1^ Total polyphenol content is expressed as gallic acid equivalents (GAE). ^2^ Total flavonoid content is expressed as quercetin equivalent (QE).

**Table 2:** The UPLC-MS/MS data of the polyphenolic compounds in Ulmi.

Peak No.	Rt (min)	Formula	Compound	[M + H]^+^	MS/MS
1	0.91	C_5_H_9_NO_2_	Proline	116	70
2	5.15	C_28_H_38_N_2_O_4_	Cephaeline	467	165
3	6.19	C_30_H_26_O_12_	Procyanidin B2	579	427, 409, 289
4	6.47	C_15_H_14_O_6_	Catechin	291	139, 123
5	7.92	C_15_H_14_O_6_	Epicatechin	291	249, 139, 123
6	10.60	C_19_H_18_O_11_	Mangiferin	423	273
7	12.04	C_21_H_20_O_12_	6-hydroxyluteolin 7-O-glucoside	465	345, 327, 315, 303
8	13.84	C_15_H_20_O_3_	Atractylenolide III	249	231

**Table 3:** Binding energy analysis of ALDH2 Ulmi compound interactions via molecular docking.

Binding Ligand	Interacting Residues	Binding Energy (kcal/mol)
Proline	MET 174, TRP 177, PHE 465	−4.8
Cephaeline	ALA 81, ARG 84, TYR 139, HIS 140, GLY 141, LYS 142, THR 143, ARG 155, THR 185	−7.9
Procyanidin B2	ILE 166, TRP 168, LYS 192, GLU 195, GLN 196, LYS 352	−9.5
Catechin	ILE 166, TRP 168, LYS 192, GLY 245, SER 246, PHE 401	−7.9
Epicatechin	PRO 167, GLU 195, GLN 349, PHE 401	−8.2
Mangiferin	ARG 84, HIS 140, GLY 141, LYS 142, ARG 155, HIS 156, THR 185	−8.2
6-hydroxyluteolin 7-O-glucoside	ARG 77, ALA 81, ARG 84, TYR 139, THR 185	−8.9
Atractylenolide III	THR 39, VAL 40	−7.4

## Data Availability

The original contributions presented in this study are included in the article. Further inquiries can be directed to the corresponding authors.

## Abbreviations

The following abbreviations are used in this manuscript:

AD	Atopic dermatitis
DNCB	2,4-Dinitrochlorobenzene
HaCaT	Human keratinocyte cell line
SKH-1	Hairless mouse strain
ALDH2	Mitochondrial aldehyde dehydrogenase 2
ROS	Reactive oxygen species
4-HNE	4-Hydroxynonenal
TNF-α	Tumor necrosis factor-alpha
IFN-γ	Interferon-gamma
NF-κB	Nuclear factor kappa B
JAK	Janus kinase
STAT	Signal transducer and activator of transcription
iNOS	Inducible nitric oxide synthase
COX-2	Cyclooxygenase-2
MTT	3-(4,5-dimethylthiazol-2-yl)-2,5-diphenyltetrazolium bromide
LC-QTOF-MS/MS	Liquid chromatography–quadrupole time-of-flight tandem mass spectrometry
IHC	Immunohistochemistry
H&E	Hematoxylin and eosin
DCF-DA	2′,7′-dichlorofluorescin diacetate
Dexa	Dexamethasone
SDS-PAGE	Sodium dodecyl sulfate-polyacrylamide gel electrophoresis
TPC	Total polyphenol content
TFC	Total flavonoid content
GAE	Gallic acid equivalent
QE	Quercetin equivalent
